# P38 MAP kinase inhibitors as potential therapeutics for the treatment of joint degeneration and pain associated with osteoarthritis

**DOI:** 10.1186/1476-9255-5-22

**Published:** 2008-12-04

**Authors:** Kimberly K Brown, Sandra A Heitmeyer, Erin B Hookfin, Lily Hsieh, Maria Buchalova, Yetunde O Taiwo, Michael J Janusz

**Affiliations:** 1 Procter & Gamble Pharmaceuticals, Inc. 8700 Mason-Montgomery Rd., Mason, OH 45040-9462, USA

## Abstract

**Background:**

Evaluate the potential role of p38 inhibitors for the treatment of osteoarthritis using an animal model of joint degeneration (iodoacetate-induced arthritis) and a pain model (Hargraeves assay).

**Methods:**

P38 kinase activity was evaluated in a kinase assay by measuring the amount of phosphorylated substrate ATF2 using a phosphoATF2 (Thr^71^) specific primary antibody and an alkaline phosphate coupled secondary antibody and measuring the OD at 405 nm. TNFα and IL-1β secretion from LPS stimulated THP-1 monocytic cells and human peripheral blood mononuclear cells were measured by ELISA. Rats treated with vehicle or p38 inhibitor were injected intra-articularly in one knee with iodoacetate and damage to the tibial plateau was assessed from digitized images captured using an image analyzer. The effect of p38 inhibitors on hyperalgesia was evaluated in rats given an intraplantar injection of carrageenan and 4 h later the paw withdrawal time to a radiant heat source was measured.

**Results:**

SB-203580 and VX-745 are both potent inhibitors of p38 with IC_50_s of 136 ± 64 nM and 35 ± 14 nM (mean ± S.D.), respectively. Similarly, SB-203580 and VX-745 potently inhibited TNF release from LPS stimulated human THP-1 cells with IC_50_s of 72 ± 15 nM; and 29 ± 14 nM (mean ± S.D.) respectively. TNF release from LPS stimulated human peripheral blood mononuclear cells was inhibited with IC_50_s 16 ± 6 nM and 14 ± 8 nM, (mean ± S.D.) for SB-203580 and VX-745 and IL-1 was inhibited with IC_50_s of 20 ± 8 nM and 15 ± 4 nM (mean ± S.D.), respectively. SB-203580 and VX-745 administered orally at a dose of 50 mg/kg resulted in the significant (p < 0.05) inhibition of joint degeneration in the rat iodoacetate model of 45% and 31%, respectively. SB-203580 demonstrated a dose related inhibition of joint degeneration of 30, 25, 12 and 8% at 50, 25, 10 and 5 mg/kg p.o. b.i.d. in the rat iodoacetate model. Similarly, both p38 inhibitors significantly (p < 0.05) attenuated the pain response (paw withdrawal time) in the Hargraeves hyperalgesia assay when administered orally at 30, 10 and 3 mg/kg.

**Conclusion:**

SB203580 and VX-745 demonstrated attenuation of both cartilage degeneration and pain in animal models and suggest that p38 inhibitors may be a useful approach for the treatment of osteoarthritis.

## Background

Osteoarthritis (OA) is a common rheumatic disease that is characterized by a progressive loss of articular cartilage. Cartilage degeneration results from an imbalance between anabolic and catabolic processes due to the dedifferentiation and apoptosis of chondrocytes and increased synthesis of matrix degrading proteinases [1]. There is increasing evidence that inflammation plays an active role in pathophysiology of osteoarthritis [2]. Proinflammatory cytokines are secreted from the inflamed synovium and from activated chondrocytes. Cytokines such as interleukin 1 beta (IL-1β) and tumor necrosis factor alpha (TNFα) upregulate numerous cytokines from chondrocytes and synoviocytes as well as prostaglandin E_2 _and proteinases such as the matrix metalloproteinases (MMPs) and aggrecanases [3-5]. The aggrecanses and the matrix metalloproteinases are thought to mediate the structural degradation of cartilage in OA [2].

Cytokines may also play an important role in driving the primary symptom of the degenerative process of OA, pain. Inflammatory cytokines such as IL-1β [6] and TNFα [7,8] have been shown to modulate pain responses in animal models and may be important in the initiation and perpetuation of neuropathic pain. Pretreatment of rats before spinal nerve ligation with the TNF antagonist etanercept (Enbrel^®^) or cytokine inhibition by the p38 inhibitor SB-203580 demonstrated similar degrees of inhibition of mechanically induced allodynia [9]. SB 203580 was also shown to attenuate IL-1 induced thermal hyperalgesia in rats when administered intrathecally [10]. These data suggest that cytokine inhibition may be useful for treating the pain associated with OA.

Monoclonal anti-TNF therapies such as infliximab (Remicade^®^) and adalimumab (Humira^®^), the TNF receptor fusion protein etanercept (Enbrel^®^), and the soluble IL-1 receptor anakinra (Kineret^®^) have proven to be effective for the treatment of a number of inflammatory diseases including rheumatoid arthritis and inflammatory bowel disease [11-16]. However, these biological cytokine inhibitors have not been widely evaluated in clinical trials for OA due to a potentially poor risk to benefit ratio and the fact that these drugs are very expensive and need to be administered parenterally.

One way to approach cytokine inhibition is with low molecular weight orally active inhibitors that block cytokine signaling pathways such as the p38 MAPK pathway [17]. The MAPKs operate as a series of kinase modules beginning with the MAPK kinase kinases (MKKKs), which phosphorylate MAPK kinases (MKKs), which ultimately phosphorylate MAP kinases (MAPK) [1]. There are 3 MAPK families, the extracellular-regulated protein kinases (ERK), the c-Jun NH2-terminal kinase (JNK) and p38 [17]. The p38 family has four members: α and β which are 75% homologous and γ and δ that are more distantly related [17]. P38 can upregulate cytokine production by several mechanisms such as direct phosphorylation of transcription factors such as AP-1 [18], or by stabilization and increased translation of mRNAs containing 3' untranslated region AU-rich elements (AREs) by phosphorylation of ARE binding proteins [18].

Small molecule p38 MAPK inhibitors have been demonstrated to attenuate the synthesis of inflammatory cytokines and MMPs [17]. SB 203580 [19-21] and VX-745 [22] are both potent inhibitors of p38α and β but not γ or δ. The specificity of a number of widely used p38 inhibitors has been more completely described recently versus large panels of kinases [23,24]. SB-203580 has good selectivity for p38α and β over the majority of kinases in a panel of over 300 kinases but does inhibit some MAPKs such as JNK 3 with an IC_50 _of about 100 nM [23,24]. VX-745 is more specific but does inhibit some tyrosine kinases with IC_50_s 10–100 fold higher than for p38 [23,24].

Numerous p38 inhibitors have been evaluated in animal models of rheumatoid arthritis [25,26] but little work has been done in experimental models of OA. One study has demonstrated a significant reduction in cartilage destruction and osteophyte formation in rabbits receiving an anterior cruciate ligament transection treated with the MEK-1/2 inhibitor PD 198306 [27]. The present study describes the effect of two well known p38 inhibitors on cartilage degradation in the iodoacetate model of joint degeneration and inhibition of hyperalgesia in the Hargraeves model. These data suggest that p38 inhibitors may be beneficial in the treatment of both joint degeneration and pain associated with OA.

## Materials and methods

### P38 kinase inhibitors

The p38 kinase inhibitors SB 203580 and VX-745 were synthesized by the chemistry department at Procter & Gamble Pharmaceuticals. The structures of the synthesized compounds were verified by nuclear magnetic resonance, mass spectroscopy and elemental analysis. The purity of the compounds was > 99% as determined by high performance liquid chromatography.

### Kinase assay procedure

P38 kinase (Upstate Biotechnology, Charlottesville, VA) was assayed in triplicate in a kinase buffer containing 25 mM HEPES, 25 mM β-glycerophosphate, 25 mM MgCl_2_, 0.1 mM Na_3_VO_4_, 2 mM DTT, and 50 μM ATP, in the presence or absence of various concentrations of inhibitor (6.4, 16, 40, 100 or 250 nM) in 96-well microtiter plates. The substrate ATF2 was used at 50 ng/reaction (coated onto plates by overnight incubation at 4°C). The reaction was carried out at 37°C for 1 h. Phosphorylated ATF2 was detected using a phosphoATF2 (Thr71) specific primary antibody (Cell Signaling) that was then followed by ALP-conjugated goat anti-rabbit IgG (Jackson Immune Research). The OD was taken at 405 nm with a reference at 490 nm.

### TNFα ELISA on culture supernatants

Duplicate cultures of human monocytic cells (THP-1) cells (2.0 × 10^5^/well) were incubated for 15 min in the presence or absence of various concentrations of inhibitor (8, 40, 125, 200, 500, 1000 or 2000 nM in RPMI-1640 with 2 mM glutamine, 10 mM HEPES, 1 mM sodium pyruvate, 10% fetal calf serum, and 0.05 mM 2-β-mercaptoethanol) before the stimulation of cytokine release by the addition of lipopolysaccharide (LPS, 1 μg/ml, E. coli 055:B5, Sigma/Aldrich, St Louis, MO, < 3% protein impurities by Lowry assay). The amount of TNF-α released was measured 4 h later using an ELISA (R&D Systems, Minneapolis, MN). The viability of the cells after the 4 h incubation was measured using the CellTiter96 Aqueous non radioactive cell proliferation assay (Promega Co., Madison, WI). The CellTiter96 Aqueous non radioactive cell proliferation assay measures cellular dehydrogenase activity as a surrogate of cellular viability by assaying the reduction of the tetrazolium compound (3-(4,5-dimethylthiazol-2-yl)-5-(3-carboxymethoxyphenyl)-2-(4-sulfophenyl)-2H-tetrazolium to formazan which is detected by a spectrophotometer at 490 nM. The viability of the THP-1 cells was > 95%.

### Inhibition of TNF-α and IL-1-β release from LPS-stimulated human peripheral blood mononuclear cells (PBMCs)

Human PBMCs from 3 healthy volunteers were isolated from 60 ml of heparinized human blood by gradient centrifugation at 400 × G for 35 min at 25°C on Ficoll-Hypaque gradients (Sigma Chemical Co, St Louis, MO). The mononuclear cells were collected from the gradient, were washed 3 times by centrifugation in Hanks balanced salt solution, counted in a hemocytometer and resuspended in RPMI 1640 medium (Gibco, Grand Island, N.Y.) containing 1% ITS supplement (insulin, transferrin, selenous acid, bovine serum albumin and linoleic acid). Duplicate cultures of human PBMCs (2.0 × 10^5^/well) were incubated for 15 minutes in the presence or absence of various concentrations of inhibitor (16, 80, 400 or 2000 nM) in the RPMI 1640 medium described above before the stimulation of cytokine release by the addition of LPS (1 μg/ml). The amount of TNF-α and IL-1β released was measured 18 h later by ELISA (R&D Systems, Minneapolis, MN). The viability of the human PBMCs was > 90%.

### Animals

Sprague-Dawley male rats weighing 220–230 grams (Harlan, Indianapolis, IN) were housed singly in wire cages in sanitary ventilated animal rooms with controlled temperature, humidity and regular light cycles. Rodent chow (Ralston-Purina, Richmond, IN) and water were available *ad libitum*. Animals were acclimated for at least one week before use.

All animal studies described in this report were conducted in compliance with the US Animal Welfare Act, the rules and regulations of the State of Ohio Departments of Health, and in accordance with the Procter& Gamble company policy of research involving animals with strict oversight for care and welfare. For details of the policy please contact the Procter & Gamble Company.

### Induction of iodoacetate-induced arthritis

Arthritis was induced by a single intraarticular injection of iodoacetate into the knee joint of rats anesthetized using (3:1) CO_2_/O_2_. A 10 mg/ml concentration of monosodium iodoacetate (MIA) (Aldrich Chemical, Milwaukee, WI) was prepared using injectable saline as the vehicle. After appropriate anesthesia each rat was positioned on its back and the left leg was flexed 90 degrees at the knee. The patellar ligament was palpated below the patella and the injection was made into this region. Each rat received 0.025 ml intra-articular injection into the left knee using a glass gas tight syringe with a 27 gauge 1/2 inch needle. Care was taken not to advance the needle in too far into the cruciate ligaments.

After injection of iodoacetate rats were treated orally b.i.d. with either vehicle (0.5% carboxymethylcellulose/0.5% Tween 20), VX 745 (50 mg/kg) or SB203580 (5, 10, 25 or 50 mg/kg) as a suspension in 0.5% carboxymethylcellulose/0.5% Tween 20 for 3 weeks. There were 15 rats in each treatment group. Upon termination of the study (3 weeks) the left knees of the euthanized animals were disarticulated and the tibial plateau imaged using an Optimas image analyzer. The tibial plateau was used for image analysis because it provided a relatively flat surface compared with the femoral condyles, allowing the image analysis camera to focus on the entire cartilage surface. The severity of damage in the magnified images was assessed by three independent observers in a blinded manner using a scale of increasing severity (0 = normal; 4 = maximum severity) as described previously [28].

### Hargraeve's model of hyperalgesia

Sprague-Dawley male rats weighting 100–150 grams were housed two per shoebox cage in sanitary, ventilated animal rooms with controlled temperature, humidity and regular light cycles were used. Rodent chow and water were allowed *ad libitum*. Animals were acclimated for one week before use.

On the first day of the study, each animal was acclimated to test equipment and thermal hyperalgesia was determined using the Hargreaves Plantar Device (infrared radiant heat source), to establish baseline paw withdrawal latency (PWL) values. The baseline PWL values were calculated as the mean of 2 pre-dose values. Animals were fasted overnight, prior to dosing. The following day animals were orally dosed (15 rats per group) with vehicle (0.5% carboxymethylcellulose/0.5% Tween 80), indomethacin (positive control 10 mg/ml) or test compound (VX-745 or SB-203580 at 3, 10 or 30 mg/kg). Thirty minutes after dosing, each animal was anesthetized using CO_2_/O_2 _(3:1) and received an intra plantar injection of 0.100 ml of a 1.2% solution (w/v) of Carrageenan Viscarin GP 109 and returned to his cage to recover. Four hours post injection; the left hind limb of each rat was assessed for the thermal hyperalgesia. The animals were placed into the Hargreaves Plantar Device once again to determine response to the heat stimulus. Three responses were recorded and the final two were averaged to determine the response at the end of the study.

### Statistical analysis

The change of paw withdrawal latency (PWL) for vehicle and drug treatment groups in the Hargraeve's assay was calculated. Statistical comparisons between treatment groups were made using analysis of covariance (ANCOVA). The percent reduction in iodoacetate induced knee degeneration in treated rats was compared to vehicle treated animals and was analyzed using the Cochran-Mantel-Haenszel test.

## Results

### SB-203580 and VX -745 are potent inhibitors of p38, TNFα and IL-1β in vitro

The in vitro potencies of SB-203580 and VX-745 for p38 and cytokine inhibition were evaluated in vitro before testing in the rat iodoacetate model of cartilage degeneration. Both SB-203580 and VX-745 were potent inhibitors of p38 with IC_50_s of 136 ± 64 nM (mean ± S.D., n = 3) and 35 ± 14 nM (mean ± S.D., n = 8), at 50 μM ATP concentrations respectively (Table 1). Similarly, SB-203580 and VX-745 potently inhibited TNF release from LPS stimulated human THP-1 cells with IC_50_'s of 72 ± 15 nM; (mean ± S.D., n = 65) and 29 ± 14 nM; (mean ± S.D., n = 3) respectively. TNF release from LPS stimulated human peripheral blood mononuclear cells was inhibited with IC_50_s 16 ± 6 nM and 14 ± 8 nM, (mean ± S.D., n = 3) for SB-203580 and VX-745 and IL-1 was inhibited with IC_50_s of 20 ± 8 nM and 15 ± 4 nM (mean ± S.D., n = 3), respectively (Table 1).

**Table 1:** In vitro inhibitory effects of SB-203580 and VX-745 on p38 and cytokine release

IC_50 _(nM)				
**Compound**	**P38α^a^**	**TNFα^b ^(THP-1 cells)**	**TNFα^c ^(PBMC)**	**IL-1β^c ^(PBMC)**

SB-203580	136 ± 64	72 ± 15	16 ± 6	20 ± 8

VX-745	35 ± 14	29 ± 14	14 ± 8	15 ± 4

^a^Human p38α kinase was assayed using ATF2.

^b^TNFα release from the human monocytic cell line THP-1 was measured by ELISA 4 h after stimulation with LPS.

^c^TNFα and IL-1β release from human peripheral blood mononuclear cells was measured by ELISA 16 h after stimulation with LPS. All data are expressed as the mean ± S.D.

### P38 inhibitors attenuate joint degeneration in the rat iodoacetate model

**Figure 1:**
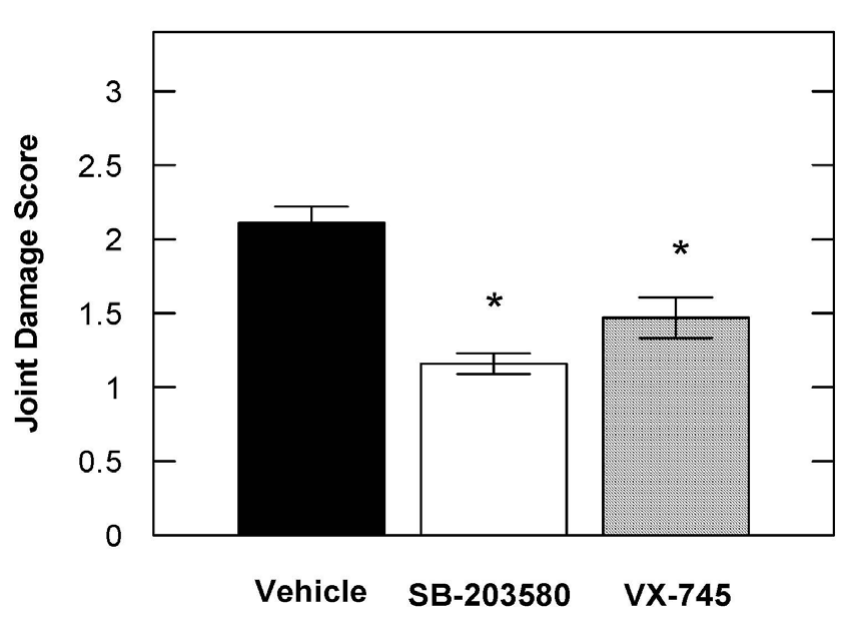
**The p38 inhibitors SB-203580 and VX-745 significantly inhibited the severity of iodoacetate-induced knee degeneration in rats.** The severity of knee degeneration in rats injected with 0.25 mg of iodoacetate was evaluated 3 weeks after treatment with either vehicle (0.5% carboxymethylcellulose/0.5% Tween 20), 50 mg/kg bid of SB-203580 or VX-745 orally. The data are expressed as the mean ± S.E.M. from 15 rats per treatment group. * denotes P < 0.05.

**Figure 2:**
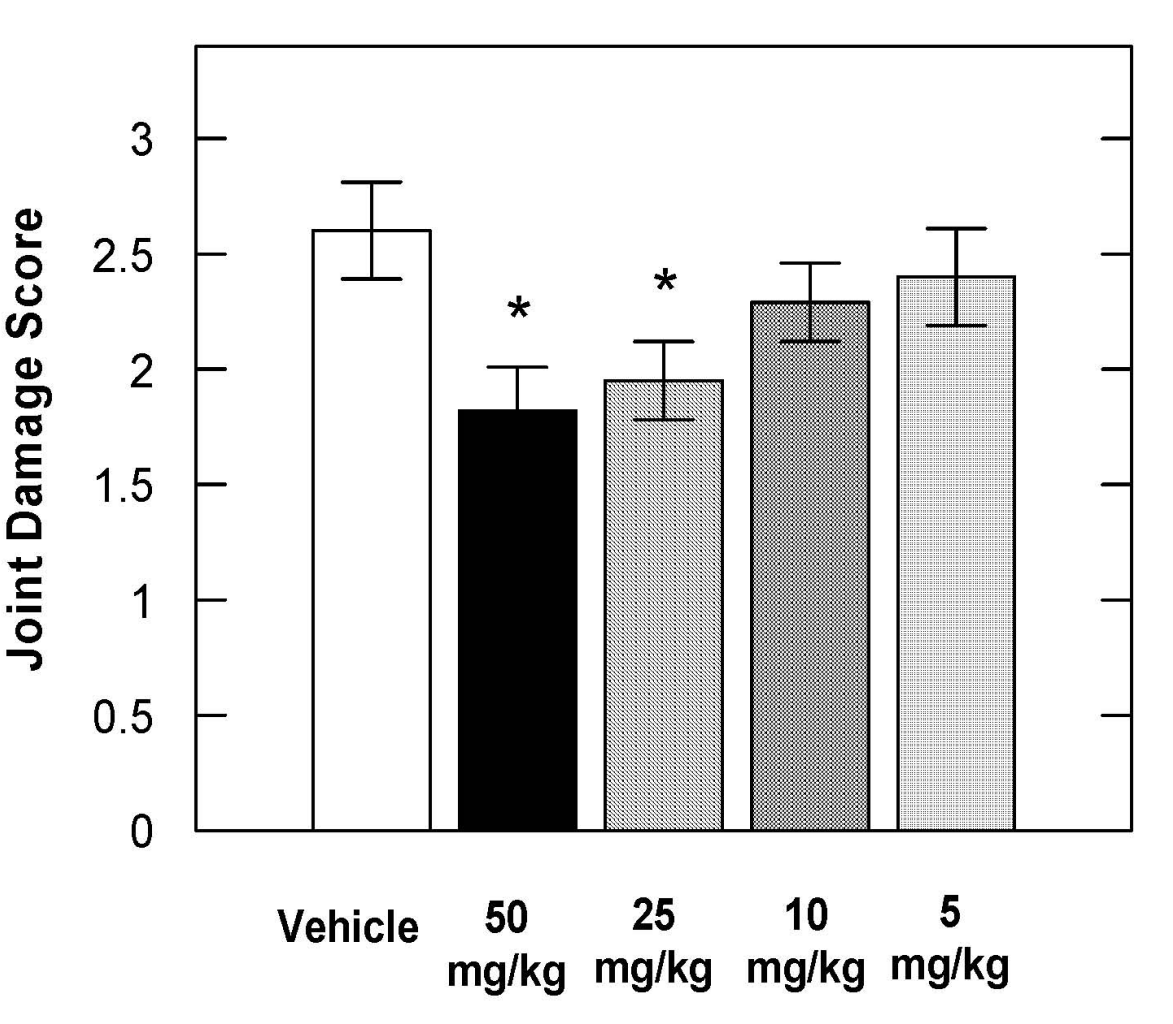
**SB-203580 inhibited the severity of iodoacetate-induced knee degeneration in rats in a dose related manner.** The severity of knee degeneration in rats injected with 0.25 mg of iodoacetate was evaluated 3 weeks after treatment with vehicle or various doses of SB-203580. The data are expressed as the mean ± S.E.M. from 15 rats per treatment group. * denotes P < 0.05.

The oral administration of SB-203580 or VX-745 (50 mg/kg b.i.d.) to rats that had received a single injection of sodium iodoacetate into the left knee joint resulted in statistically significant inhibition of knee degeneration of 45% and 31% respectively, compared to vehicle treated control animals (Figure 1). SB-230580 was further evaluated in the rat iodoacetate model in a dose response experiment. SB-203580 administered orally inhibited iodoacetate induced joint degeneration in the rat by 30, 25, 12 and 8% at 50, 25, 10 and 5 mg/kg compared to vehicle treated animals (Figure 2).

### P38 inhibitors attenuate hyperalgesia in rats

The p38 inhibitors were evaluated for their ability to inhibit a hyperalgesic response in rats using the Hargraeves model [29]. Rats were given an oral dose of vehicle, SB-203580 or VX-745 and 30 minutes later one paw of each rat received an intraplantar injection of carrageenan. The time to paw withdrawal to an infrared heat source was measured four hours later. Both SB-203580 (Table 2) and VX-745 (Table 3) significantly increased the time to paw withdrawal compared to vehicle treated animals in a dose related manner when administered orally at 30, 10 and 3 mg/kg.

**Table 2:** Inhibitory effect of SB-203580 on hyperalgesia in the Hargraeves model

COMPOUND	PAW WITHDRAWAL TIME PRE-DRUG TREATMENT (Seconds)	PAW WITHDRAWAL TIME POST-DRUG TREATMENT (Seconds)
Vehicle	10.7 ± 0.6	8.0 ± 0.8

Indomethacin (10 mg/kg)	11.1 ± 0.6	14.5 ± 0.6*

SB-203580 (30 mg/kg)	10.8 ± 0.9	12.2 ± 1.2*

SB-203580 (10 mg/kg)	11.1 ± 0.6	11.8 ± 1.4*

SB-203580 (3 mg/kg)	12.0 ± 0.7	10.9 ± 1.2*

The data are presented as the time for paw withdrawal from the heat source in seconds and are the mean ± S.E.M. from 10 rats per treatment group. The asterisk denotes a statistically significant difference (p < 0.05) between the vehicle and drug treatment groups.

**Table 3:** Inhibitory effect of VX-745 on hyperalgesia in the Hargraeves model

COMPOUND	PAW WITHDRAWAL TIME PRE-DRUG TREATMENT (Seconds)	PAW WITHDRAWAL TIME POST-DRUG TREATMENT (Seconds)
Vehicle	11.5 ± 0.8	7.4 ± 0.5

Indomethacin (10 mg/kg)	12.3 ± 0.9	16.3 ± 1.2*

VX-745 (30 mg/kg)	10.6 ± 0.4	14.9 ± 0.6*

VX-745 (10 mg/kg)	11.7 ± 0.8	12.1 ± 0.7*

VX-745 (3 mg/kg)	12.0 ± 0.7	9.4 ± 1.0*

The data are presented as the time for paw withdrawal from the heat source in seconds and are the mean ± S.E.M. from 10 rats per treatment group. The asterisk denotes a statistically significant difference (p < 0.05) between the vehicle and drug treatment groups.

## Discussion

The degeneration of cartilage that occurs during osteoarthritis is the result of biochemical and mechanical factors. Proinflammatory cytokines play a major role in inducing proteinases that are capable of degrading the aggrecan and collagen components of cartilage [2,30]. Cytokines such as IL-1β have also been shown to inhibit cartilage matrix synthesis [31,32]. The result is an imbalance favoring catabolism over anabolism and a net loss of cartilage matrix. In addition, inflammatory cytokines such as IL-1β [6] and TNFα [7,8] have been shown to modulate pain responses in animal models. Therefore, the inhibition of proinflammatory cytokines may provide an important therapeutic approach for the treatment of OA.

Clinical trial data on cytokine inhibitors for the treatment of OA is limited. Diacerein, is a compound that inhibits IL-1β production in vitro [33,34] and has been evaluated in several clinical trials for the treatment of OA. Diacerein has been shown to significantly decrease OA symptoms [35,36] and to have structure modifying effects [37]. A 3 month pilot study of the TNF antagonist adalimumab in 12 patients with erosive OA did not significantly improve the signs and symptoms of the disease [38] but this study was uncontrolled, small and of short duration.

MAPKs such as p38 have been widely pursued targets for the inhibition of cytokines for the treatment of inflammatory diseases [39]. Although p38 inhibitors have been extensively studied in animal models inflammatory arthritis [25,26] there has been little work in models of osteoarthritis. In the present study we have demonstrated that the p38 inhibitors SB203580 and VX-745 inhibit joint degeneration in an animal model of osteoarthritis and are analgesic in an inflammatory pain model. The inhibitory effect of the p38 inhibitors on joint degeneration in the iodoacetate model in the present study is in the range of the inhibitory effect of MMP inhibitors tested in this model [28]. Although the effects of p38 inhibitor in OA models has not been previously reported the interleukin converting enzyme inhibitor pralnacasan was shown to reduce joint damage in a surgically induced model and a spontaneous model of OA in mice [40] demonstrating a benefit of cytokine inhibition. Similarly, intra-articular injection of IL-1RA attenuated the development of cartilage lesions and metalloproteinase expression in canine models of OA [41]. These animal studies demonstrate that some of the inflammatory mediators affected by p38 play an important role in the cartilage degeneration observed in a number of different animal models of OA. However, it is difficult to translate the effects observed in these animal models into a clinical benefit in human OA as there are currently no approved disease modifying drugs for the treatment of OA.

A number of protein kinases including the MAPKs have been implicated in the induction and maintenance of pain sensitization. P38 is activated in spinal microglia and contributes to the development and maintenance of neuropathic pain by inducing the synthesis of inflammatory cytokines and other neuroactive molecules [42]. Administration of the p38 inhibitor SB203580 intrathecally prevents spinal nerve ligation-induced mechanical allodynia [43]. The p38 inhibitor R-130823 has been reported to have an analgesic effect in chronic pain in the rat adjuvant arthritis model [44]. This effect may involve the bradykinin B1 receptor as a p38 has been shown to mediate hyperalgesia via the bradykinin B1 receptor in adjuvant induced arthritis [45]. These data support the use of p38 inhibition to directly affect the pain of osteoarthritis.

## Conclusion

In the present study, p38 inhibitors had a therapeutic benefit in both models of joint degeneration and in hyperalgesia. These data suggest that p38 inhibitors may be useful for the treatment of both joint degeneration and pain associated with OA.

## Abbreviations

TNFα: Tumor necrosis factor alpha; IL-1α: interleukin 1 α; OA: osteoarthritis; p38: p38 mitogen activated kinase; PBMCs: peripheral blood mononuclear cells.

## Competing interests

All of the authors were employees of Procter & Gamble Pharmaceuticals during the performance of these studies and received 100% of their compensation from the company.

## Authors' contributions

KKB, SAH and EBH performed all of the in vivo studies and analyzed the data. LH performed all the in vitro studies and analyzed the data. MB designed and prepared the formulations for the p38 inhibitors tested in vivo. YOT contributed to experimental design and data analysis. MJJ designed in vitro and in vivo studies analysed the data and wrote the manuscript.

## References

[B1] Berenbaum F (2004). Signaling transduction: target in osteoarthritis. Curr Opin Rheumatol.

[B2] Pelletier JP, Martel-Pelletier J, Abramson SB (2001). Osteoarthritis, an inflammatory disease. Potential implication for the selection of new therapeutic targets. Arthritis Rheum.

[B3] Abramson SB, Yazici Y (2006). Biologics in development for rheumatoid arthritis: Relevance to osteoarthritis. Adv Drug Deliv Rev.

[B4] Malemud CJ (2004). Cytokines as therapeutic targets for osteoarthritis. BioDrugs.

[B5] Tortorella MD, Malfait AM, Arner E (2001). The role of ADAM-TS4 (aggrecanase-1) and ADAM-TS5 (aggrecanase-2) in a model of cartilage degradation. Osteoarthritis Cartilage.

[B6] Samad TA, Moore KA, Sapirstein A, Billet S, Allchorne A, Poole S, Bonventre JV, Woolf CJ (2001). Interleukin-1β-mediated induction of COX-2 in the CNS contributes to inflammatory pain hypersensitivity. Nature.

[B7] Wagner R, Myers RR (1996). Endoneurial injection of TNF-alpha produces neuropathic pain behaviors. NeuroReport.

[B8] Sommer C, Schafers M, Marziniak M, Toyka KV (2001). Etanercept reduces hyperalgesia in experimental painful neuropathy. J Peripher Nerv Syst.

[B9] Schafers M, Svensson CI, Sommer C, Sorkin LS (2003). Tumor necrosis factor-α induces mechanical allodynia after spinal nerve ligation by activation of p38 MAPK in primary sensory neurons. J Neurosci.

[B10] Sung CS, Wen ZH, Chang WK, Chan KH, Ho ST, Tsai SK, Chang YC, Wong CS (2005). Inhibition of p38 mitogen-activated protein kinase attenuates interleukin-1β-induced thermal hyperalgesia and inducible nitric oxide synthase expression in the spinal cord. J Neurochem.

[B11] Lipsky PE, Heijde D van der, St Claire EW, Furst DE, Breedveld FC, Kalden JR, Smolen JS, Weisman M, Emery P, Feldmann M, Harriman GR, Maini RN (2000). Infliximab and methotrexate in the treatment of rheumatoid arthritis. N Engl J Med.

[B12] Targan SR, Hanauer SB, van Deventer SJH, Mayer L, Present DH, Braakman T, DeWoody KL, Schaible TF, Rutgeerts PJ (2008). A short-term study of chimeric monoclonal antibody cA2 to tumor necrosis factor α for crohn's disease. N Engl J Med.

[B13] Hanauer SB, Feagan BG, Lichtenstein GR, Mayer LF, Schreiber S, Colombel JF, Rachmilewitz D, Wolf DC, Olson A, Bao W, Rutgeerts P (2002). Maintenance infliximab for Crohn's disease: the ACCENT I randomized trial. The Lancet.

[B14] Weinblatt ME, Keystone EC, Furst DE, Moreland LW, Weisman MH, Birbara CA, Teoh LA, Fischkoff SA, Chartash EK (2003). Adalimumab, a fully human anti-tumor necrosis factor α monoclonal antibody, for the treatment of rheumatoid arthritis in patients taking concomitant methotrexate. Arthritis Rheum.

[B15] Weinblatt ME, Kremer JM, Bankhurst AD, Bulpitt KJ, Fleischmann RM, Fox RI, Jackson CG, Lange M, Burge DJ (1999). A trial of etanercept, a recombinant tumor necrosis factor receptor: Fc fusion protein, in patients with rheumatoid arthritis receiving methotrexate. N Engl J Med.

[B16] Cohen S, Hurd E, Cush J, Schiff M, Weinblatt ME, Moreland LW, Kremer J, Bear MB, Rich WJ, McCabe D (2002). Treatment of rheumatoid arthritis with anakinra, a recombinant human interleukin-1 receptor antagonist, in combination with methotrexate. Arthritis Rheum.

[B17] Saklatvala J (2004). The p38 MAP kinase pathway as a therapeutic target in inflammatory disease. Curr Opin Pharmacol.

[B18] Ashwell JD (2006). The many paths to p38 mitogen-activated protein kinase activation in the immune system. Nature Reviews.

[B19] Kumar S, McDonnell PC, Gum RJ, Hand AT, Lee JC (1997). Novel homologues of CSBP/p38 MAP kinase. Biochem Biophys Res Comm.

[B20] Wadsworth SA, Cavender DE, Beers SA, Lalan P, Schaffer PH, Malloy EA, Wu W, Fahmy B, Olini GC, Davis JE, Pellegrino-Gensey JL, Wachter MP, Siekierha JJ (7657). RWJ 6 a potent, orally active inhibitor of p38 mitogen activated protein kinase. J Pharmacol Exp Ther.

[B21] Davies SP, Reddy H, Caivano M, Cohen P (2000). Specificity and mechanism of action of some commonly used protein kinases inhibitors. Biochem J.

[B22] Salituro F VX-745, a selective p38 inhibitor. American Chemical Society 33rd Middle Atlantic Regional Meeting.

[B23] Fabian MA, Biggs WH, Treiber DK, Atteridge CE, Azimioara MD, Benedetti MG, Carter TA, Ciceri PT, Edeen PT, Floyd M, Ford JM, Galvin M, Gerlach JL, Grotzfeld RM, Herrgard S, Insko DE, Insko MA, Lai AG, Lelias JM, Mehta SA, Milanov ZV, Velasco AM, Wodicka LM, Patel HK, Zarrinkar PP, Lockhart DJ (2005). A small molecule-kinase interaction map for clinical kinase inhibitors. Nat Biotechnol.

[B24] Karaman MW, Herrgard S, Treiber DK, Gallant P, Atteridge CE, Campbell BT, Chan KW, Ciceri P, Davis MI, Edeen PT, Faraoni R, Floyd M, Hunt JP, Lockhart DJ, Milanov ZV, Morrison MJ, Pallares G, Patel HK, Pritchard S, Wodicka LM, Zarrinkar PP (2008). A quantitative analysis of kinase inhibitor selectivity. Nat Biotechnol.

[B25] Badger AM, Bradbeer JN, Votta B, Lee JC, Adams JL, Griswold DE (3580). Pharmacological profile of SB 20 a selective inhibitor of cytokine suppressive binding protein/p38 kinase, in animal models of arthritis, bone resorption, endotoxin shock and immune function. J Pharmacol Exp Ther.

[B26] Badger AM, Griswold DE, Kapadia R, Blake S, Swift BA, Hoffman SJ, Stroup GB, Webb E, Rieman DJ, Gowen M, Boehm JC, Adams JL, Lee JC (2235). Disease-modifying activity of SB 24 a selective inhibitor of p38 mitogen-activated protein kinase, in rat adjuvant arthritis. Arthritis Rheum.

[B27] Pelletier JP, Fernandes JC, Brunet J, Moldovan F, Schrier D, Flory C, Martel-Pelletier J (2003). In vivo selective inhibition of mitogen-activated protein kinase kinase 1/2 in rabbit experimental osteoarthritis is associated with a reduction in the development of structural changes. Arthritis Rheum.

[B28] Janusz MJ, Hookfin EB, Heitmeyer SA, Woessner JF, Freemont AJ, Hoyland JA, Brown KK, Hsieh LC, Almstead NG, De B, Natchus MG, Pikul S, Taiwo YO (2001). Moderation of iodoacetate-induced experimental osteoarthritis in rats by matrix metalloproteinase inhibitors. Osteoarthritis Cartilage.

[B29] Hargreaves K, Dubner R, Brown F, Flores C, Joris J (1988). A new sensitive method for measuring thermal nociception in cutaneous hyperalgesia. Pain.

[B30] Goldring MB (2000). Osteoarthritis and cartilage: The role for cytokines. Curr Rheumatol Rep.

[B31] Nietfeld JJ, Wilbrink B, Helle M, van Roy JL, den Otter W, Swaak AJ, Huber-Bruning O (1990). Interleukin-1-induced interleukin-6 is required for the inhibition of proteoglycan synthesis by interleukin-1 in human articular cartilage. Arthritis Rheum.

[B32] Geng Y, Valbracht, Lotz M (1996). Selective activation of the mitogen-activated protein kinase subgroups c-Jun NH2 terminal kinase and p38 by IL-1 and TNF in human articular chondrocytes. J Clin Invest.

[B33] Martel-Pelletier J, Mineau F, Jolicoeur FC, Cloutier JM, Pelletier JP (1998). In vitro effects of diacerhein and rhein on interleukin 1 and tumor necrosis factor-α systems in human osteoarthritic synovium and chondrocytes. J Rheumatol.

[B34] Moldovan F, Pelletier JP, Jolicoeur FC, Cloutier JM, Martel-Pelletier J (2000). Diacerhein and rhein reduce the ICE-induced IL-1β and IL-18 activation in human osteoarthritic cartilage. Osteoarthritis Cartilage.

[B35] Pellitier JP, Yaron M, Haraoui B, Cohen P, Nahir MA, Choquette D, Wigler I, Rosner IA, Beaulieu AD, the Diacerein Study Group (2000). Efficacy and safety of diacerein in osteoarthritis of the knee: a double-blind, placebo controlled trial. Arthritis Rheum.

[B36] Pavelka K, Trc T, Karpas K, Vitek P, Sedlackova M, Vlasakova V, Bohmova J, Rovensky J (2007). The efficacy and safety of diacerein in the treatment of painful osteoarthritis of the knee. Arthritis Rheum.

[B37] Dougados M, Nguyen M, Berdah L, Mazieres B, Vignon E, Lequesne M, for the ECHODIAH Investigators Study group (2001). Evaluation of the structure-modifying effects of diacerein in hip osteoarthritis: ECHODIAH, a three-year, placebo controlled trial. Arthritis Rheum.

[B38] Genevay S, Stringelin S, Gabay C (2004). Efficacy of etanercept in the treatment of acute, severe sciatica: a pilot study. Ann Rheum Dis.

[B39] Schindler JF, Monahan JB, Smith WG (2007). P38 pathway kinases as anti-inflammatory drug targets. J Dent Res.

[B40] Rudolphi K, Gerwin N, Verzijl N, Kraan P van der, Berg W van der (2003). Pralnacasan, an inhibitor of interleukin-1β converting enzyme, reduces joint damage in two murine models of osteoarthritis. Osteoarthritis Cartilage.

[B41] Caron JP, Fernandes JC, Martel-Pelletier J, Tardif G, Mineau F, Geng C, Pelletier JP (1996). Chondroprotective effect of intraarticular injections of interleukin-1 receptor antagonist in experimental osteoarthritis. Suppression of collagenase-1 expression. Arthritis Rheum.

[B42] Ji RR, Suter MR (2007). p38 MAPK, microglial signaling, and neuropathic pain. Molecular Pain.

[B43] Jin SX, Zhuang ZY, Woolf CJ, Ji RR (2003). P38 mitogen-activated protein kinase is activated after a spinal nerve ligation in spinal cord microglia and dorsal root ganglion neurons and contributes to the generation of neuropathic pain. J Neurosci.

[B44] Wada Y, Nakajima-Yamada T, Yamada K, Tsuchida J, Yasumoto T, Shimozato T, Aoki K, Kimura T, Ushiyama S (0823). R-13 a novel inhibitor of p38 MAPK, ameliorates hyperalgesia and swelling in arthritis models. Eur J Pharmacol.

[B45] Ganju P, Davis A, Patel S, Nunez X, Fox A (2001). p38 stress-activated protein kinase inhibibtor reverses bradykinin B1 receptor-mediated component of inflammatory hyperalgesia. Eur J Pharmacol.

